# Exploring knowledge, perceptions, and practices of antimicrobials, and their resistance among medicine dispensers and community members in Kavrepalanchok District of Nepal

**DOI:** 10.1371/journal.pone.0297282

**Published:** 2024-01-19

**Authors:** Sabina Marasini, Sudim Sharma, Anjali Joshi, Surakshya Kunwar, Roshan Kumar Mahato, Archana Shrestha, Biraj Karmacharya

**Affiliations:** 1 Department of Public Health, Kathmandu University School of Medical Sciences, Dhulikhel, Nepal; 2 Faculty of Public Health, Mahidol University, Bangkok, Thailand; 3 Department of Chronic Disease Epidemiology, Yale School of Public Health, New Haven, Connecticut, United States of America; 4 Institute for Implementation Science and Health, Kathmandu, Nepal; University of Hail, SAUDI ARABIA

## Abstract

**Background:**

Inappropriate use of antimicrobials is a global public health issue, particularly in developing countries, including Nepal, where over-the-counter sales and self-medication of antimicrobials are common.

**Objectives:**

To explore knowledge, perceptions, and practices of antimicrobials and their resistance among medicine dispensers and community members in Nepal.

**Methods:**

The study was conducted in three rural and five urban municipalities of the Kavrepalanchok district from May 2021 to August 2021. Data were collected using two qualitative approaches: In-Depth Interviews (IDIs) and Focus Group Discussions (FGDs). Data were analyzed using thematic analysis that combined deductive and inductive approaches to identify codes under pre-specified themes.

**Results:**

A total of 16 In-depth interviews with medicine dispensers and 3 focus group discussions with community members were conducted. Knowledge regarding antimicrobial resistance varied among dispensers. Those with a prior educational background in pharmacy often had good knowledge about the causes and consequences of antimicrobial resistance. Meanwhile, consumers were less aware of antimicrobial resistance. Community members perceived antimicrobials as effective medicines but not long-term solution for treating diseases. They reported that dispensing without a prescription was common and that both consumers and dispensers were responsible for the inappropriate use of antimicrobials. On the contrary, several dispensers said that self-medication was common among the consumers, especially among more educated groups. The medicine dispensers and consumers expressed concerns about the weak enforcement of policies regarding pharmacy drug use and dispensing practices.

**Conclusion:**

Promoting and strengthening the appropriate use of antimicrobials among medicine dispensers and community members is crucial. Bold policies and collective implementation of regulations could help combat antimicrobial resistance.

## Introduction

Antimicrobial resistance (AMR) is widely considered a global threat and is accelerated by the misuse and overuse of antimicrobials [1]. An estimated one million people died from antimicrobial-resistant diseases between the years 2014 and 2016, while the current forecast suggests that resistance would cause 300 million premature deaths and economic losses of US$100 trillion by 2050 [2]. The widespread and inappropriate use of antimicrobials has led to AMR, a condition in which pathogenic bacteria develop resistance to the specific antimicrobial prescribed against it [3, 4].

AMR is ubiquitous in lower-resource settings, such as Nepal. Nepal’s health service is predominantly shaped by the private sector, with around 70% of all health expenditures attributable to out-of-pocket spending [5, 6]. The country’s basic healthcare delivery is facilitated by unqualified conventional practitioners and medicine distributors, lacking official professional qualifications or trainings. This situation led to irrational dispensing of antimicrobials, often in situations when they are unnecessary [7, 8]. The pharmacies and medical stores in Nepal operate under the regulations outlined in the Drug Act of 1978 [9]. According to this act, pharmacists (who have completed a 4-year bachelor’s degree in pharmacy after 12 years of schooling), assistant pharmacists (with a 3-year diploma in pharmacy after 10 years of schooling), or pharmacy “professionals” (with a 3-day orientation) can run a pharmacy/medical store by registering with the Department of Drug Administration (DDA) [5, 10]. Despite these regulations, many patients rely on health assistants, pharmacists and informal providers as their primary source of allopathic healthcare because of low doctor-patient ratios and insufficient distributions of qualified medical doctors [11, 12].

In Nepal, antimicrobial resistance has considerably worsened in recent years. Antimicrobials are commonly used as growth promoters in livestock and agriculture. Moreover, self-medication practices, lack of point-of-care diagnostic services, and easy access to over-the-counter (OTC) antimicrobials (without prescriptions) significantly contribute to AMR [12]. Additionally, inadequate knowledge about the rational use of antimicrobials and a fragile drug regulatory mechanisms exacerbate the resistance crisis in Nepal [13, 14].

Several studies have explored different aspects of antimicrobial use in Nepal, including self-medication practices, public awareness concerning antimicrobial use and resistance, and antimicrobial use in primary health care [15–18]. However, there remains a paucity of exploration into the perspectives held by medicine dispensers at community pharmacies and community members regarding the use of antimicrobials and AMR. Therefore, this study aimed to understand the knowledge and perceptions of the medicine dispensers and consumers regarding antimicrobial use and resistance in the Kavrepalanchok District in central Nepal.

## Materials and methods

### Design

We pursued an exploratory qualitative study involving In-Depth Interviews (IDIs) with Medicine dispensers and Focus Group Discussions (FGDs) with community members in the Kavrepalanchok District of Nepal. The primary objective was to explore the knowledge and perception regarding antimicrobial use and resistance. Qualitative methods were chosen for their capacity to delve into context and meaning, capturing perspectives of the involved participants [19].

### Setting

We conducted the study in both urban and rural municipalities of the Kavrepalanchok District of Nepal. Kavrepalanchok district lies in the Bagmati province and has a population of 364,039 [20]. The district consists of 13 municipalities, of which six are urban and seven are rural [21]. For our study, we selected five urban municipalities- Dhulikhel, Banepa, Panauti, Paanchkhal, and Mandan Deupur and three rural municipalities- Chauri Deurali, Bhumlu, and Temal. Study sites were selected based on convenience and logistical feasibility.

### Sample

We recruited medicine dispensers from community pharmacies and community members from the 13 aforementioned municipalities. “Medicine dispensers” in our study refers to anyone who sells antimicrobials at a pharmacy or medical store (including qualified pharmacists with a degree or those with little or no formal pharmacy training). Dispensers working in the allopathic pharmacies were recruited for the IDIs using a mixed approach of purposive and snowball sampling. We conducted 16 IDIs with medicine dispensers at community pharmacies. Meanwhile, we recruited community members between the ages of 20–60 years to incorporate diverse perspectives from various groups and backgrounds. The sample sizes were determined based on the principle of saturation [22].

### Participant recruitment

The researcher (SM) coordinated with the District Health Office and oriented the local health coordinators about the study. The health coordinators of the local government, responsible for managing health related affairs at local level, contacted medicine dispensers for IDIs and mapped their interest in the study participation. The health coordinators provided the contact details of 7 medicine dispensers interested in the study. Then, we contacted an additional 12 participants via snowball sampling. After obtaining the list of interested participants, researcher (SM) communicated with them via telephone, outlining the study’s purpose, expectations from participants and ethical considerations emphasizing participant’s privacy, voluntary participation, the option to withdraw, and the requirement for audio-recording of interviews.

For recruitment of the participants in the FGD, we employed purposive sampling. The researcher (SM) contacted the health coordinator to explain study’s purpose, while the health coordinators approached community members to identify eligible participants from various background to capture the diverse perspectives.

### Data collection techniques

For IDIs with medicine dispensers, we met the participants on-site to explain the study’s objective and request their participation. Upon obtaining the informed consent, we conducted six face-to-face interviews in the pharmacy settings. Due to the several restrictions of the COVID-19 pandemic, we shifted to telephone interview for the remaining 10 IDIs. All the interviews were conducted over a period of three months, from April 2021 to June 2021. Three participants had to drop out as they were unable to allocate time. The interviews were audio-recorded with the consent of the participants. The interviews ranged between 23 to 65 minutes.

For FGDs with community members, we contacted the health coordinators and outreach centers affiliated with Dhulikhel Hospital across different municipalities of Kavrepalanchok to select the participants and the locations for discussion. With the help of these community representatives, we decided on a location and invited participants by phone. Trained moderators conducted FGDs in private settings close to the community members. The groups varied in size from 6 to 10 participants. The FGDs were audio-recorded with consent from participants and ranged between 40 to 75 minutes.

### Data collection tools

We used semi-structured guides to facilitate data collection, using separate guides for IDI and FGD. The guides were formulated by reviewing literature to determine themes and consulting with experts for probing questions in Nepali language [23–27]. Before initiating the study, the interview guide and FGD guide were pre-tested among medicine dispensers and community people from Kathmandu (a city nearby the study site). The pre-test data were not included in the analysis. During the IDIs, we asked questions such as: “What do you know about antimicrobials?”, “What are the things that you follow while dispensing the antimicrobials?”, “What do you understand by antimicrobial resistance?”, and “What do you think about the current use of antimicrobials?” We also probed participants with further questions when necessary. During the focus group discussion, we asked questions such as: “What do you do when you get sick?”, “What do you know about antimicrobials?”, “Where do you get antimicrobials?”, “How do you decide the use of antimicrobials?”, “What do you know about antimicrobial resistance?” The data was collected following an iterative process where certain revisions were made on interviews based on the insights from the prior interviews.

### Data management and analysis

To ensure anonymity, we assigned an alpha-numeric code to each participant and de-identified their transcripts. We used thematic analysis, initially developing a deductive codebook based on the interview guides, anticipated responses from the literature review, and pre-tested data [28]. As the analysis progressed, we added additional codes inductively emerged from the data. The interviews were transcribed verbatim into Nepali and were coded. Meaningful units, like quotes, were condensed and labelled with codes. Similar codes were grouped into categories, which were further organized under overarching themes.

### Ethical consideration

The Kathmandu University Institutional Review Committee (KUIRC) granted ethical approval (IRC: 124/20) for conducting the study. For the in-person interviews, we obtained written informed consent from individuals who agreed to participate in the study. Meanwhile, for telephone interviews, oral consent was obtained after explaining the purpose of the study, and confidentiality was explained to participants. We then created the list of IDI participants who consented to participate in our study. Following the initial call, we allocated specific time slot for each participant at their convenience. We also obtained consent to record the discussion. Most importantly, participation was voluntary, and confidentiality was maintained in this study.

## Results

We carried out 16 IDIs with medicine dispensers and 3 FGDs, engaging a total of 25 participants. Table 1 provides an overview of the background characteristics of the participants.

**Table 1:** Characteristics of the study participants.

IDI Participants (n = 16)	
**Educational Background**	
Pharmacy related degree (Bachelor in Pharmacy)	4 (25%)
Degree other than pharmacy (Health Assistant, Community medicine Assistant, Auxiliary Nurse Midwives, and others)	12 (75%)
**Years of experience as medicine dispenser (mean±S.D)**	16.1 ± 10.8
**Registration of Pharmacy**	
Pharmacy registered under DDA	15 (94%)
Pharmacy not registered under DDA	1 (6%)
FGD Participants (n = 25)	
**Gender**	
Male	11 (44%)
Female	14 (56%)
**Occupation**	
Businessman	6 (24%)
Office staff	3 (12%)
Teacher	10 (40%)
Female Community Health Volunteer	3 (12%)
Homemakers	3 (12%)

Among the 16 medicine dispensers, only 4 (25%) held a pharmacy degree. A majority of the pharmacies (94%) were registered under the DDA. Among the 25 FGD participants, 56% were females. The community members from various occupation groups- teacher, businessman, female community health volunteers, homemakers and office staff were included in the discussion.

### Medicine dispensers’ knowledge of antimicrobials use

Most of the study participants described antimicrobials as antibacterial medicines used to suppress the growth of bacteria. They described antimicrobials as not a regular drug but one that should be taken for a specific duration. Additionally, participants highlighted that antimicrobials are not effective for treating viral infections.

*“Antimicrobials are the medicines which kill or slow down bacteria*, *but they do not work against viral infections*.*”*

*(P15)*

Most dispensers described symptoms as the basis for deciding when to use antimicrobials, while a few referenced lab reports and prescriptions for their decisions. Meanwhile, most of the dispensers described the current use of antimicrobials as irrational. Some of the participants related the irrational use of antimicrobials with the insufficient health care resources.

*“75% of the current use is inappropriate and this is because of the lack of fully trained health workers and lack of awareness of the consumers*……*the consumers prefer to go to the places where double triple medicines are dispensed for fast recovery*.*”*

*(P9)*

#### Medicine dispensers’ practices related to antimicrobial use

The participants stored their medicines based on dates (expiry date) and location considering temperature and protection from sunlight to maintain their integrity. While dispensing the antimicrobials, most of the dispensers gave information regarding the dose, duration, and possible side effects, such as gastrointestinal upset and diarrhea. Some also mentioned the practice of inquiring about allergies and other medical conditions before dispensing the antimicrobials.

*“We tell the patients about the dose and duration of the antimicrobials and tell them to complete the dose even if they feel okay after a few doses*.*”*

*(P8)*

Dispensing antimicrobials without a prescription was a common practice among dispensers. They mentioned patients’ signs and symptoms as the basis for antimicrobial dispensing, stating no viable alternative. Additionally, dispensers often supplied antimicrobials upon patient’s specific requests, assuming that if they know the name of specific antimicrobial, they are knowledgeable about its usage.

*“Actually*, *it is wrong to give antimicrobials without a prescription*, *but we do not have other choices here*. *Not everyone can reach hospitals and laboratories*. *People compare the medication of a pharmacy with hospitals after tests*. *They think it is cheaper to get medicines from here (pharmacy) than going to hospitals for tests*.*”*

*(P7)*

#### Medicine dispensers’ knowledge of antimicrobial resistance

The medicine dispensers showed varying degree of knowledge about antimicrobial resistance (AMR). The dispensers with pharmacy background demonstrated comprehensive understanding regarding the causes and consequences of AMR. However, some participants displayed limited awareness, with a few associating allergic reactions with AMR. Participants also recounted their experiences of AMR. The medicine dispensers with longer experience in dispensing medicines had perceived changes in the sales of antimicrobials, attributing this change to increased consumer literacy, resulting in increased self-medication practices.

*“Ciprofloxacin was the go-to drug for enteric fever*, *but people misused it by taking it whenever they had fever and keep coming back with complaints of recurring fever*. *As we had no other alternative*, *we had to send the patient to the hospital*, *where they found Ciprofloxacin resistance*.*”*

*(P13)*

*“Earlier people were hesitant to take antimicrobials*. *But nowadays*, *they think themselves that they should take antimicrobials whenever they feel sick*.*”*

*(P11)*

Many participants perceived underuse, overuse, self-medication, and non-compliance with the prescribed doses as the primary drivers of increasing AMR. They highlighted unnecessary antimicrobial usage and excessive dispensing by non-pharmacy personnel at community pharmacies as contributors to AMR.

*“I think self-medication practice is one of the reasons behind antimicrobial resistance*. *In Nepal*, *antimicrobials are easily available OTC*. *Nowadays*, *people search on the internet for medicines*. *They buy azithromycin based on google search and take one strip without understanding the proper use*.*”*

*(P13)*

*“The consequences could be that antimicrobials might not work as they should*. *For instance*: *Amoxicillin used to be effective but now it needs to be paired with clavulanic acid to be effective*.*”*

*(P5)*

The participants said that there is not a single entity or individual accountable for AMR. They expressed a collective belief that the government had a major responsibility for increased AMR and held the potential to exert a substantial influence in resolving the issue of AMR.

*“I blame the health workers in 90% of the cases as they sell 2/3 types of antimicrobials just for making money*. *Many dispensers are not trained or trained for 5–6 months (insufficient) and do not even know the right dosage of antimicrobials*.*”*

*(P9)*

*“There is health post nearby us where there is constant shortage of antimicrobials and there is no laboratory facility*. *Only if they had a lab in that health post*, *it would be so helpful as the health post could do tests and prescribe medicines*.*”*

*(P7)*

#### Medicine dispensers’ perceptions about regulatory issues

The majority of participants referred to the disclaimer in the medicine strip “to be dispensed by physician only”, as the main guideline for dispensing antimicrobials. Many dispensers admitted either not knowing or forgetting the regulations due to the absence of training or review meetings specifically addressing antimicrobials. One of the participants mentioned the existence of guidelines delineating different levels/hierarchy of health professionals for dispensing antimicrobials.

*“There are rules which have assigned specific antimicrobials for use at different levels of healthcare*. *Different lists are allowed for Auxiliary Health Worker (AHW)*, *Bachelor of Medicine and Bachelor of Surgery (MBBS) and Doctor of Medicine (MD)*.*”*

*(P9)*

Many participants said that the rules were not effective and existed on paper only, not in practice. They highlighted implementation issues as a significant concern. Additionally, one participant remarked on the impracticality of obtaining prescription for every antimicrobial, citing the scarcity of healthcare personnel in rural areas as a hindrance to rule enforcement.

*“I don’t think it is practical*. *Imagine needing Azithromycin- can you always get a prescription for it*? *In rural areas without doctors*, *is it possible to get prescriptions dispensing antimicrobials there*?*”*

*(P4)*

Most of the participants emphasized the pivotal role of government in ensuring the efficacy of the rules. They identified several areas where government needs to enforce strictness in regulations, such as enhancing medical facilities in government health centers, ensuring the presence of medical doctors at primary care level, enforcing stringent regulations in community pharmacies mandating the presence of pharmacists in their establishment and conducting regular supervision and routine continuing medical education of the medicine dispensers.

*“The reason why the rules do not work well is because of lack of a health workforce*. *The government sends antimicrobials to the health post*, *but we do not have doctors there*, *just paramedics*. *So*, *it is a challenge for the government whether to allow paramedics to handle antimicrobials*.*”*

*(P13)*

*“To minimize antimicrobial resistance*, *the most important thing is to raise awareness among consumers*, *because if patients are themselves not aware*, *they will not take medications (complete doses) even if doctors prescribe it*. *This knowledge should be included in health subject’s curriculum in school*. *Other than that*, *awareness activities through television*, *radio*, *health units through documentaries or other means (might work)*.*”*

*(P13)*

The interval for supervision by Department of Drug Administration (DDA) was reported to be different. The participants said that concerning antimicrobials, the date and storage location of antimicrobials was supervised, the same as for other regular medicines, but not their dispensing.

*“During supervision of pharmacy*, *the supervisors look at the records of sales of narcotic drugs but I do not remember being asked about the record of sales of antimicrobials*.*”*

*(P13)*

#### Medicine dispensers’ roles in the rational use of antimicrobials

Most of the dispensers expressed concern about the current irrational use of antimicrobials. A majority of the dispensers believed that their major roles as medicine dispensers were proper counselling and promoting the rational use of antimicrobials, i.e. dispensing them only when necessary. Some participants mentioned a feeling of social pressure while working in a community pharmacy, sometimes being compelled to perform activities out of their scope. They highlighted the challenges of meeting societal demands, indicating difficulty in convincing the patients, especially from lower socio-economic status, to complete the full course of antimicrobials due to their higher cost compared to other medicines. Moreover, one participant cited the challenge of upholding ethical principles as a hurdle in their role as a medicine dispenser.

*“Sometimes we have to do things we don’t know*, *for example*, *we have to learn to do the dressing*, *suturing*. *Sometimes people tell us to administer saline*, *which we are not supposed to do but have to do it*.*”*

*(P4)*

*“People hesitate to pay NRs*. *50 as doctor’s fee in community pharmacies*, *yet they are willing to pay NRs*. *300 at a hospital in the city*. *There are different problems at community level*.*”*

*(P10)*

Many issues were identified by medicine dispensers at community pharmacies regarding antimicrobial use. The misuse of a pharmacist’s license and the pharmacies run by a person without a degree in pharmacy were identified as problems. The easy availability of antimicrobials and competition among pharmacies led to over-dispensing antimicrobials. Similarly, dispensers also underscored issues such as lack of consumer awareness and absence of laboratory facilities in health centers, particularly in rural areas. Additionally, they highlighted the prevalence of self-medication among individuals, where some people independently decide to take medications without consulting physicians and stop treatment once symptoms subside.

*“The Nepalese government mandates that pharmacist must hold a Bachelor of Pharmacy degree and a license to operate a pharmacy*. *However*, *it is noticeable that pharmacists rent out their licenses for a fee*, *allowing other individuals without a (requisite) degree to operate pharmacies*.*”*

*(P10)*

*“The Community Medicine Assistant (CMA) and Auxiliary Nurse Midwife (ANM) at community pharmacies misuse the antimicrobials*…*They prioritize fast recovery of patients to maintain steady patient flow*. *Over-dispensing of antimicrobials has become a marketing tactic in community pharmacies*.*”*

*(P12)*

*“In Nepal*, *antimicrobials are widely available as the OTC drugs and are easily accessible in pharmacies*. *For instance*, *if someone requests for Azithromycin*, *they have option to choose between 500mg or 1000 mg*.*”*

*(P13)*

Current needs felt by the medicine dispensers were trainings and updates on new issues. Along with this, they advocated for rigorous monitoring and supervision of pharmacists’ licenses. Many emphasized the role of having pharmacist present in pharmacies, considering it a potential solution to various challenges. One participant said that along with other measures, raising consumer awareness to induce behavior change could effectively control the irrational use of antimicrobials.

*“Even the medicine dispensers who have been running pharmacies for years might not realize that many antimicrobials are now resistant*. *An up-to-date information regarding the antimicrobial resistance is needed for the medicine dispensers too*, *be it through formal or informal trainings or other ways*.*”*

*(P13)*

*“The main concern is that DDA is silent (inactive) despite an evident absence of pharmacists in pharmacies*.*”*

*(P12)*

*“The community-based local pharmacies are the first option for community people to get antimicrobials*. *If we could regulate these community pharmacies through law*, *it could help control the inappropriate use of antimicrobials*.*”*

*(P12)*

*“To improve the current irrational use*, *antimicrobials should not be dispensed without a prescription of a doctor*….. *we cannot be sure that the patients adhere to the 5–7 days dose of antimicrobials*. *So*, *raising awareness among the patients is very important and it can be done starting in school*.*”*

*(P13)*

#### Community members’ perceptions about access to healthcare

The majority of the participants mentioned hospitals, health posts and primary health care centers, and medical pharmacies as their first choice for healthcare. The participants sought allopathic care for communicable as well as non-communicable diseases. All the participants said that they try home remedies for sickness and go to health centers if the disease persists. The participants said that they commonly take paracetamol or Ibuprofen for fever and pain relief before going to the hospital or other health centers.

*“It depends on the nature of disease and the easy access to any of the health centers be it health post or primary health center or medical store (pharmacy)*.*”*

*(FGD2*,*P4)*

#### Community members’ knowledge regarding antimicrobials

The participants described antimicrobials as the medicines to treat infections. Some participants described antimicrobials as comparatively strong and expensive medicines with a fixed dose. Antimicrobials were believed to be effective medicines for the fast recovery of diseases. The participants believed that antimicrobials helped them to get better quickly.

Although the participants believed antimicrobials to help in fast recovery, they also acknowledged their effects and potential long-term impact on the body.

*“Antimicrobials are used when a normal medicine does not show effect*. *So*, *I think the antimicrobials make our body weaker*.*”*

*(FGD2*; *P2)*

#### Community members’ perceptions about availability of antimicrobials

The pharmacies or medical stores were the primary location for purchasing antimicrobials, as per the participants. They noted that the antimicrobials are available at a discounted price at health posts compared to the pharmacies.

*“Other medicines are available in the grocery store but antimicrobials are not available there*.*”*

*(FGD2*; *P3)*

*“In the health post*, *the medicines are less expensive*; *we get 10% discount on medicines in health post*.*”*

*(FGD2*; *P2)*

Meanwhile, some participants said that they showed prescriptions from doctors to get the medicines in pharmacies. It is a common practice among the physicians to make weekly/monthly visits in some pharmacies where they attend to patients and offer specialized services. The participants reported that they visited these specialist physicians at the pharmacies and acquired the medications based on their advice.

Additionally, the participants added that the treatments at pharmacies/medical store depend on the individual preferences. They have the option to either consult a doctor or seek advice from the dispenser. In cases where they opt for dispenser’s guidance, medicines are provided without a prescription.

*“They do not look for prescription (written)*; *oral conversation works*.*”*

*(FGD1*; *P3)*

*“We go and ask for medicine to a dispenser*, *to avoid the doctor fee*. *In such cases also we don’t need a prescription*.*”*

*(FGD3*; *P9)*

#### Community members’ perceptions towards antimicrobials

The decision to use antimicrobials depended on the doctor’s prescription and advice. Common practices included consuming paracetamol for fever, ibuprofen for pain relief, and pantoprazole for gastritis. However, participants noted that antimicrobials were not taken without professional guidance or prescription.

*“When medicine helped us to get better*, *we used to take that medicine without consulting doctor*. *But nowadays*, *the trend of taking antimicrobials after lab test and (doctor’s) prescription is increasing*.*”*

*(FGD1*; *P10)*

Participants believed antimicrobials to be effective medicines with fast action. However, some also pointed out that though antimicrobials are fast in action but might not be a sustainable solution for treatment of infections.

*“It has been a trend to use antimicrobials if we feel sick*. *The antimicrobials help us to get better but there are its negative impacts (side effects) too*.*”*

*(FGD3*; *P9)*

Additionally, the participants supposed that counselling impacts patient’s psychology and helps in recovery. Most participants acknowledged that doctors and dispensers did good counselling but that the duration was dependent on the volume of patients they attend. The participants highlighted a significant difference in the antimicrobial dispensing practice between private pharmacies and government health centers where the private pharmacies tend to dispense more antimicrobials to gain more profit.

*“When we go to the medicine counter without doctor’s prescription*, *the counter is always crowded and patients become confused*. *Often people don’t purchase full medicines*; *in the crowd the medicines are left or misplaced*.*”*

*(FGD3*; *P2)*

*“There is a difference between health centers and medical store*. *In health centers*, *the health workers usually do not give antimicrobials and primarily prescribe basic medicines*. *But in medical store (pharmacies)*, *they dispense more antimicrobials to make profit because antibiotics are expensive (with higher profit margin)*.*”*

*(FGD2*; *P4)*

#### Community members’ practices related to antimicrobials use

The practice of self-medication with antimicrobials was reportedly very low among the participants. The participants said that though they consumed other medicines like paracetamol, pantoprazole and ibuprofen from self-medication, they refrained self-medicating the antimicrobials. Instead, they mentioned purchasing medicines without a prescription but sought advice from doctors or other healthcare professionals.

#### Community members’ knowledge of antimicrobial resistance

Overall, the participants demonstrated a low level of knowledge concerning AMR. After a brief overview of AMR, some participants related it to instances where the dosage of antimicrobials are increased.

Though the participants were not familiar with the term “antimicrobial resistance”, they were aware about the inappropriate use of antimicrobials. They had observed a higher prevalence of the inappropriate use in private settings such as pharmacies, clinics, compared to the government health centers like health post, primary healthcare centre (PHCC) and government hospitals. They emphasized that AMR is not solely the responsibility of a single entity but rather stems from various levels. The participants believed that the government should take more responsibility for controlling the misuse of antimicrobials.

*“In the hospital they give us antimicrobial by looking at the disease*. *But in medical store (pharmacy)*, *they give us antimicrobials be it necessary or not necessary*. *If possible*, *we should not go to medical stores*.*”*

*(FGD3*; *P2)*

*“Patients themselves teach the doctors*, *telling them that the medicines provided did not work and demand for something stronger*. *This is not a good practice*.*”*

*(FGD1*; *P3)*

Improving the public awareness of the rational use of antimicrobials is crucial in the current context where misuse prevail within community. Participants noted the scarcity of antimicrobial-related programs at the community level. They expressed concerns regarding AMR and proposed potential solutions:

- Conducting educational programs in the community to raise awareness about AMR, emphasizing the consumer responsibility in adhering to prescribed doses and durations.
- Restricting the dispensing of antimicrobials to prescription-only by the pharmacists.
- Implementing periodic supervision ad monitoring of pharmacies by government bodies.

The participants acknowledged that while awareness is valuable, it is insufficient on its own; access to quality healthcare and medicines is equally vital for the rational use of antimicrobials.

*“Raising public awareness and good practices should go together*. *It is not just about understanding the right use*, *it is also about putting it into practice*…..*so*, *it is interconnected and needs to progress parallelly*.*”*

*(FGD2*; *P4)*

The participants reported that pharmacies are believed to be profit-making businesses rather than being service-oriented. The presence of a non-pharmacist or a person from other educational background at pharmacies was observed by many participants and they noted this to be a problem during the discussion too.

*" Running a pharmacy is like a family business where one person of the family starts the pharmacy and other members join the same*. *And we all know that often the person running the pharmacy neither have knowledge nor they do have skills like the medical doctors*.*”*

*(FGD3*; *P2)*

### Summary of the findings

**Table:** 

**Findings from IDI with Medicine Dispensers**	**Findings from FGD with community members**
The medicine dispensers described antimicrobials as the antibacterial medicines used to suppress the growth of bacteria; not a regular drug but the one which should be taken for a certain duration and also mentioned that they are not used to treat viral infections. Dispensing antimicrobials without prescription was a common practice among dispensers. Many participants perceived underuse, overuse, self-medication and non-compliance with the dose as the causes of increasing AMR. The use of antimicrobials when not necessary and the over dispensing of antimicrobials by the non-pharmacy personnel at community pharmacies were considered to be the causes of AMR. The dispensers with full pharmacy training were well aware of the causes and consequences of antimicrobial resistance. The misuse of a pharmacist’s license and the pharmacies run by people without a pharmacy degree were identified as problems. The easy availability of antimicrobials and competition among pharmacies were identified as factors leading to over-dispensing of the antimicrobials. Likewise, the lack of laboratory facilities in health centers, mostly in the rural areas, were also issues raised by the medicine dispensers.	The community members described antimicrobials as medicines to treat infections; comparatively strong and expensive medicines with a fixed dose; effective medicines for fast recovery of diseases. The pharmacies or medical stores were the most common locations for buying antimicrobials. The practice of self-medication with antimicrobials was found to be very low. The participants said that though they consumed other medicines like paracetamol, pantoprazole and ibuprofen on their own but did not consume antimicrobials on their own. Knowledge regarding AMR was very low among the participants. The participants were aware about the inappropriate use of antimicrobials and reported that the inappropriate use of antimicrobials was more evident in private healthcare centers like pharmacies, clinics and comparatively less in the government health centers like health posts, primary healthcare centers, and hospitals. As reported by community members, running a pharmacy is assumed to be a profit-making business rather than service-oriented. The presence of a non-pharmacist or a person from another educational background at pharmacies was observed by many participants.

## Discussion

This study aimed to explore the knowledge and perceptions of antimicrobial use and resistance among medicine dispensers and community members in Nepal. The study highlights that the inappropriate use of antimicrobials stems from the actions of medicine dispensers and consumers. Consumers, often unaware of negative consequences and facing economic constraints, take below-prescribed doses. Dispensers, due to limited diagnostic facilities, may rely on clinical judgement for dispensing the antimicrobials. A weak regulatory environment, profit-driven motive, and competition in pharmacies also contribute to over-dispensing. Similar findings concerning demand and supply factors contributing to inappropriate antimicrobial use have been reported in other studies conducted in low-and middle-income countries (LMICs) [17, 26, 29–33].

The level of knowledge on antimicrobials significantly influences the purchasing behaviors and also contributes to the misuse of antimicrobials [34]. Along with the poor knowledge and awareness of the risks associated with the dangers and consequences of irrational use of antimicrobials, easy access to OTC drugs, lack of awareness, and limited access to healthcare also contribute to the inappropriate use of antimicrobials [30, 35]. Patient’s expectations for fast recovery influence doctor’s decision to prescribe strong antimicrobials. FGD participants revealed that patients often request strong antimicrobials themselves. Meanwhile, the doctors find it challenging to refuse prescriptions, particularly for children, well-known clients, and the individuals they like [36].

The inappropriate sales of antimicrobials by unqualified health professionals is a significant contributor to increasing AMR. In Nepal, apart from public and private health facilities, there are many formal and informal pharmacies. Most pharmacies are managed by non-pharmacists, who operate under someone else’s license or even without a license [37]. The Drug Act 1978 and Nepal Pharmacy Council mandate the antimicrobials to be sold with prescriptions and dispensed by qualified and registered pharmacists [9, 38]. These regulations are largely disregarded with poor compliance to the Good Pharmacy Practice (GPP) [39]. Many studies in Nepal have consistently documented the OTC sales of antimicrobials by dispensers, bypassing the regulations [40, 41]. This practice is often driven by patient’s demand for fast recovery and the dispenser’s aim to maintain good relationship with consumers. Moreover, competition between pharmacies can influence the prescribing behaviors. Dispensers might feel societal pressure to cure the patient quickly to meet their expectations and uphold their reputation in the community. Since antimicrobials are one of the profitable drugs, some dispensers might dispense them excessively to maximize their profits [42]. As found in our study, renting a pharmacy license to a non-pharmacist to own and operate a pharmacy is widespread, despite the existence of regulations against such practices [42].

Healthcare providers, particularly in rural areas, face challenges and limitations in delivering services, primarily due to scarcity of resources for investigation [43, 44]. This expression was echoed by many dispensers in this study. Consequently, the dispensers rely on their judgement to provide antimicrobials, often when they are not clinically necessary. In Nepal, only 25% of the public health facilities have access to laboratory services and those that do, have limited tests available [45]. The laboratory facilities are predominantly available in urban hospitals. As a result, individuals from rural areas constrained by economic limitations cannot access high-quality care and depend on the dispensers and their clinical judgement.

The absence of a robust antimicrobial policy and a weak regulatory framework within the healthcare system contributes to the inappropriate use of antimicrobials [38]. In Nepal, the Drug Act 1978 stands as the primary legislative instrument governing drug usage [9]. Concerning AMR, the Government of Nepal has introduced the National Antimicrobials Treatment Guidelines and initiated a laboratory-based surveillance system since 2005 [46]. However, our study revealed that many dispensers were unaware of these guidelines, and many felt that the regulatory system lacked efficiency. While a medical prescription is mandatory for purchasing antimicrobials in Nepal, dispensing without one remains a common practice [46]. Operating healthcare services without license is illegal in Nepal, yet several unregistered pharmacies are operating in the rural areas [47]. This reflects a notable weakness in Nepal’s health system’s regulatory infrastructure. Many dispensers in our study highlighted this issue, citing it as a significant problem and a contributing factor for AMR.

Health professionals in LMICs often encounter challenges due to inadequate on-the-job trainings [48]. This concern was raised by the dispensers in our study. Research indicates that insufficient training can lead to inappropriate prescribing or dispensing of medicines [36]. Effective monitoring of prescription and dispensing can enhance the clinical decision-making [49]. In our study, the dispensers expressed concern over irregular supervision, which predominantly focused on narcotics and lacked antimicrobial monitoring, thereby fostering their inappropriate use. The DDA oversees the registration and monitoring of pharmacies, including prescription audits. However, these audits and processes are not regular [50]. Participants too noted that while supervision occurred, strict actions were seldom taken in case of regulation violation.

In our study, the FGD participants were unaware that purchasing antimicrobials without a prescription could contribute to AMR, thereby reducing their efficacy. These findings align with prior studies from Nepal, that reported a lack of understanding related to antimicrobials and resistance among patients and the general population [17, 45, 51–53].

AMR stands as a global public health concern primarily driven by the inappropriate use of antimicrobials. To address this crisis, the WHO in 2015 endorsed a Global Action Plan comprising of five strategic objectives. Among these, three of the strategies directly correlate with the findings of our study: Strengthening knowledge through surveillance and research, optimizing the use of antimicrobial agents, and improving the awareness and understanding of AMR [54]. Executing these strategies on a national scale holds the potential to mitigate the repercussions stemming from inappropriate use of antimicrobials. Simultaneously, improving the awareness and understanding of antimicrobial resistance must coincide with behavior change communication efforts. Tailored programs targeted at consumers should convey information regarding the issues associated with inappropriate use of antimicrobials [55].

In Nepal, pharmacies/medical stores serve as the primary source of antimicrobials for a majority of population. Solely restricting the OTC use of antimicrobials might pose significant challenge by limiting their access. Therefore, the provision of consistent training to dispensers becomes pivotal in fostering proper practice in antimicrobial dispensing [56]. Similarly, research endeavors aiming to devise effective strategies to limit the unnecessary use of antimicrobials should focus on the knowledge and behaviors of healthcare practitioners such as doctors, pharmacists, medicine dispensers, and consumers. Consequently, bridging the knowledge gap and implementing multifaceted behavior change interventions are likely to yield more effective outcomes [34].

### Implications of the study

The findings of this study unequivocally delineate an implementation gap within antimicrobial policies. While Nepal possesses established policies promoting the judicious use of antimicrobials, they contend with a frail regulatory framework and inadequate execution. This discrepancy presents a compelling area for future scholars to delve into, exploring methods to redress these issues and proffer policy recommendations. Furthermore, adopting a “One Health Approach” offers a pathway to scrutinize antimicrobial usage within animal and agriculture domains. The insights from this study hold promise in planning and developing models for behavior change communication strategies.

### Strengths and limitations

This study has several limitations. Firstly, the research was conducted during the COVID-19 pandemic, which imposed various constraints, hindering the planned execution of the study. Secondly, all three FGDs were conducted in urban settings. Consequently, the results might not be broadly generalizable to other subgroups. Thirdly, the IDIs necessitated indirect inquiries about dispensing practices to further explore participant perceptions and practices. Despite employing non-leading and non-judgmental questioning techniques, there exists the possibility of social desirability bias. Participants discussing behaviors, specifically inappropriate antimicrobial use, might have exhibited reluctance in expressing their genuine views.

Nevertheless, this study exhibits strengths, by presenting the diverse perception and practices among both dispensers and consumers. The complementary use of both IDIs and FGDs helps to compensate for the limitations of either individual. This study contributes important information regarding the perceptions on antimicrobial resistance at the community level.

## Conclusion

Rational antimicrobial prescribing and dispensing practices are necessary to reduce the alarming public health hazard of AMR. The study findings underscore that inappropriate use stems from a complex interplay of demand and supply considerations, compounded by a weak policy-regulatory environment. As such, policymakers should adopt a comprehensive, multifaceted strategy engaging stakeholders across multiple fronts to effectively tackle this issue. Since antimicrobial use and resistance are not limited to single institution or individual patient, interventions need to extend to diverse communities and encompass multiple healthcare settings. Therefore, an integrated approach comprising population-level interventions, inter-sectoral coordination, patient education initiative, robust surveillance, and monitoring emerges as imperative to prevent the growing threat of AMR.

## Supporting information

S1 Data (XLSX)

S2 Data (XLSX)

S1 File (DOCX)
