# Correction: Exploring knowledge, perceptions, and practices of antimicrobials, and their resistance among medicine dispensers and community members in Kavrepalanchok District of Nepal

**DOI:** 10.1371/journal.pone.0322320

**Published:** 2025-04-04

**Authors:** Sabina Marasini, Sudim Sharma, Anjali Joshi, Surakshya Kunwar, Roshan Kumar Mahato, Shrinkhala Shrestha, Archana Shrestha, Biraj Karmacharya

Shrinkhala Shrestha should be included in the author byline. Shrinkhala Shrestha should be listed as the sixth author, and his affiliation is 1: Department of Public Health, Kathmandu University School of Medical Sciences, Dhulikhel, Nepal. The contributions of this author are as follows: Conceptualization, Methodology, Resources, Supervision.

The correct citation is: Marasini S, Sharma S, Joshi A, Kunwar S, Mahato RK, Shrestha S, et al. (2024) Exploring knowledge, perceptions, and practices of antimicrobials, and their resistance among medicine dispensers and community members in Kavrepalanchok District of Nepal. PLoS ONE 19(1): e0297282. https://doi.org/10.1371/journal.pone.0297282
