# Supplementary material for: Exploring knowledge, perceptions, and practices of antimicrobials, and their resistance among medicine dispensers and community members in Kavrepalanchok District of Nepal
Source: PLoS One. 2024 Jan 19;19(1):e0297282. doi: 10.1371/journal.pone.0297282 (PMC10798439; doi:10.1371/journal.pone.0297282)
Supplement: S1 File — (DOCX) [file pone.0297282.s003.docx]

**ANNEX III: IDI GUIDE (ENGLISH)**

**For medicine dispensers:**

*Socio-demographic characteristics:*

Age (years)
Gender
Education
Years of experience
Job status (owner/staff)

Professional License status

Years of service at current place

**Theme 1: Knowledge of Antibiotic Use**

1.1 What do you know about antibiotics? Can you please Explain. (Probes: What types of antibiotics are available at your pharmacy? What determines the choice of antibiotic?)

1.2 What proportion of all the medicines that you sell are antibiotics? (Probes: For which illnesses do you usually sell antibiotics? Which are the most commonly sold antibiotics?)

**Theme 2. Practices**

2.1 How and where do you store your antibiotics? (Probes: What things do you consider while storing the antibiotics?)

2.2 How do you deal with the dates on the antibiotics you sell? (Probe: What do you do with drugs that have passed their expiry date? And for the drugs near expiry? How do you dispose expired antibiotics?)

2.3 What sort of information (verbal or written)/counselling regarding the proper dose/duration do you give to your customers about the antibiotics that you sell them? (Probe: What information do you give? Do they ever ask questions regarding the dose and duration of antibiotics? Do you ever ask about allergies? Or any sort of patient conditions like pregnancies and history of any chronic diseases before providing the antibiotic?

2.4 What are your opinions regarding the patient’s adherence on the dose of antibiotics? (Probe: Do you think they take the full dose? If not, why do you think this happens? Or can you share the reason behind this? Do you provide anything less than a recommended full course? If so, why and how frequently?)

2.5 What do you do when customers ask for specific antibiotic? (Probe: Do you ask your customers if they have used antibiotics before and for what conditions/diseases? Do you ask for the prescription with your customer before giving them the antibiotics? Or what do you do if they don’t have the prescription?)

2.6 What is the practice of returning unused antibiotics by customers? (Probe: If they return, how do you manage those antibiotics?)

**Theme 3. Knowledge of Antibiotic Resistance**

3.1 What do you know about antibiotic resistance? Please share (Probe: How is it caused? What are the reasons? What are the consequences?)

3.2 Have you ever encountered a patient with antibiotic resistance? Please share your experience.

3.3 Who do you think is responsible for the antibiotic resistance? (Probe: How do you see them responsible?)

**Theme 4. Regulatory Issues**

4.1 What are the regulations/standard guidelines/protocols that you have to follow in order to sell antibiotics? (Probe: regarding prescriptions, manual? How often are the guidelines updated? What challenges, if any, do you face in following these regulations/standard guidelines/protocols? What about the penalties for sellers who sell antibiotics without prescription?)

4.2. How do you solve the confusions while dispensing the antibiotics? 4.3. How do you see the current use of antibiotics? (Probe: What changes do you feel in the use of antibiotics? How do you feel about the current se, appropriate/inappropriate, how?)

4.3 What is the practice of monitoring and supervision? (Probe: Who comes for supervision? How often ae the supervisory visits done? What do they look after?)

4.4 In your opinion, how sufficient are the current government regulations to control inappropriate antibiotic use? If not, how could things be improved?

**Theme 5. Role of Pharmacist for Rational use of antibiotics**

5.1 What problems/issues do you face as a pharmacist? (Probe: societal pressure, dispensing without prescription, recommending less dose due to economic condition of customer) how do you tackle them?

5.2 What could be your role as a pharmacy person to improve practices of antibiotic use?

5.3 What do you think are the current needs of the pharmacist/medicine seller regarding the antibiotic use? (Probe: proper training, updated guidelines, monitoring and supervision)

**6.** Would you like to share your concluding remarks? Or do you like to say anything to us or about this study?

**Thank You for your responses valuable time.**

**FGD GUIDE**

Socio-Demographic Characteristics

Age (years)
Gender
Religion

Ethnicity

Education

Occupation

**Theme 1. Access to health care**

1.1 What do you do when you get sick? (Probes: What actions do you take when you are not feeling well? Where do you go for treatment? How accessible are the health centres? For what type of diseases do you seek for allopathic care?)

**Theme 2. Knowledge of Antibiotics**

2.1 What do you know about antibiotics? (Probes: What are the objectives of using antibiotics? For what type of diseases are antibiotics used ?)

2.2 How are antibiotics different from other medicines?

2.3. What could be the negative effects of antibiotics?

**Theme 3. Availability of Antibiotics**

3.1 How would you describe the availability of antibiotics? (Probe: Where and how do you get them?)

3.2. What is the practice of having prescription for antibiotics?

**Theme 4. Perceptions towards Antibiotics**

4.1 What are your perceptions toward antibiotics? (Probes: What drives your decision to take antibiotics? How effective do you find antibiotics to treat a particular disease/condition?)

4.2. Have you ever faced a situation where a doctor has not prescribed you an antibiotic and you felt you needed one? (Probes: What did you do in this case? Were you explained the reason behind this? If not, do you think an explanation would have made you more comfortable with the decision?)

4.3 What expectations do you have from healthcare workers when you are prescribed with antibiotics? (Probes: Does the doctor explain the prescription(dose/duration/follow-up/recommendations) to you? Do you know which ones are antibiotics, if any? What do you think about a doctor who doesn’t prescribe antibiotics?)

**Theme 5. Practices of Antibiotics Use**

5.1 How often do you self-medicate with antibiotics? (Probes: For what symptoms do you self-medicate with antibiotics? When was the last time? What could be the reasons for self-medicating with antibiotics?)

5.2 From who/where do you get information on self-medication with antibiotics? (Probes: What information do you get on self-medication with antibiotics?)

5.3 How well do you understand the negative consequences of antibiotic use? (Probes: Has anyone explained these to you during any consultations? How do you weigh the risks and benefits of taking antibiotics? Have you experienced any negative consequences?)

**Theme 6. Knowledge of Antibiotic Resistance**

6.1 How have you understood about antibiotic resistance? (Probes: What are its causes? Do you know anyone with antibiotic resistance in your family/community?)

6.2 Who do you think is responsible for the antibiotic resistance? (Probe: How do you see them responsible?)

6.3. How do you perceive the current use of antibiotics? (Probe: Appropriate or inappropriate, why?)

6.4 What do you think could be done to tackle this resistance?

6.5 How can the understanding of the community around the rational uses of antibiotics be improved ? (Probe: How can the risks of misusing antibiotics be explained to the community?)

Thank You!
